# Mortality analysis of adult burn patients in Uruguay

**DOI:** 10.5935/0103-507X.20200008

**Published:** 2020

**Authors:** Martín Angulo, Ignacio Aramendi, Julio Cabrera, Gastón Burghi

**Affiliations:** 1 Centro Nacional de Quemados, Cátedra de Medicina Intensiva, Facultad de Medicina, Universidad de la República - Montevideo, Uruguay.

**Keywords:** Burns/mortality, Wounds and injuries, Risk factors, Uruguay, Queimaduras/mortalidade, Ferimentos e lesões, Fatores de risco, Uruguai

## Abstract

**Objective:**

To determine the independent risk factors associated with mortality in adult burn patients.

**Methods:**

This was a retrospective, observational study performed at the *Centro Nacional de Queimados do Uruguai*. All patients with skin burns admitted to the unit since its opening on July 1, 1995 through December 31, 2018 were included. The demographic data, burn profiles, length of stay, mechanical ventilation duration and hospital mortality were studied. A multivariate logistic regression was used to identify the risk factors for mortality. The standardized mortality ratio was calculated by dividing the number of observed deaths by the number of expected deaths (according to the Abbreviated Burn Severity Index).

**Results:**

During the study period, 3,132 patients were included. The median total body surface area burned was 10% (3%-22%). The Abbreviated Burn Severity Index was 6 (4 - 7). Invasive mechanical ventilation was required in 60% of the patients for a median duration of 6 (3 - 16) days. The median length of stay in the unit was 17 (7 - 32) days. The global mortality was 19.9%. Crude mortality and standardized mortality ratio decreased from 1995 through 2018. The global standardized mortality ratio was 0.99. A need for mechanical ventilation (OR 8.80; 95%CI 5.68 - 13.62), older age (OR 1.07 per year; 95%CI 1.06 - 1.09), total body surface area burned (OR 1.05 per 1%; 95%CI 1.03 - 1.08) and extension of third-degree burns (OR 1.05 per 1%; 95%CI 1.03 - 1.07) were independent risk factors for mortality.

**Conclusion:**

The need for mechanical ventilation, older age and burn extension were independent risk factors for mortality in the burned adult Uruguayan population.

## Abstract (pt)

**Resumo:**

Determinar os fatores independentes de risco associados com mortalidade em pacientes queimados adultos.

**Métodos:**

Estudo observacional retrospectivo, conduzido no Centro Nacional de Queimados do Uruguai. Incluíram-se todos os pacientes com queimaduras da pele admitidos à unidade desde sua inauguração em 1° de julho de 1995 até o dia 31 de dezembro de 2018. Estudaram-se os dados demográficos, perfis de queimadura, tempo de permanência, duração da ventilação mecânica e mortalidade hospitalar. Utilizou-se regressão logística multivariada para identificação dos fatores de risco para mortalidade. A proporção padronizada de mortalidade foi calculada pela divisão do número de óbitos observados pelo número de óbitos previstos, segundo o *Abbreviated Burn Severity Index*.

**Resultados:**

Durante o período do estudo, incluímos 3.112 pacientes. A mediana da área total de superfície corporal queimada foi de 10% (3% - 22%). O *Abbreviated Burn Severity Index* foi de 6 (4 - 7). Foi necessária ventilação mecânica em 60% dos pacientes, com duração mediana de 6 (3 - 16) dias. O tempo mediano de permanência na unidade foi de 17 (7 - 32) dias. A mortalidade global foi de 19,9%. A mortalidade bruta e a proporção padronizada de mortalidade diminuíram entre 1995 e 2018. A proporção padronizada de mortalidade global foi de 0,99. Necessidade de ventilação mecânica (RC 8,80; IC95% 5,68 - 13,62), idade mais avançada (RC 1,07 por ano; IC95% 1,06 - 1,09), área total de superfície corporal queimada (RC 1,05 por 1%; IC95% 1,03 - 1,08) e extensão de queimaduras de terceiro grau (RC 1,05 por 1%; IC95% 1,03 - 1,07) foram fatores de risco independentes para mortalidade.

**Conclusão:**

Necessidade de ventilação mecânica, idade mais avançada e extensão da queimadura foram fatores de risco independentes para mortalidade na população adulta de pacientes queimados no Uruguai.

## INTRODUCTION

Burn injuries represent a major public health problem worldwide. In 2004, nearly 11 million people suffered burns severe enough to require medical attention.^(1)^ It is estimated that burns and fire-related injuries cause over 150,000 deaths every year.^(2)^ Older age, burn size and the presence of inhalation injury are among the most important factors associated with elevated mortality.^(3-6)^ Moreover, although most burn injuries are not fatal, in many cases, they are associated with severe and permanent sequelae and disabilities. In fact, burns represent one of the principal causes of disability-adjusted life-years in low- and middle-income countries (LMICs).

Burn epidemiology and outcomes differ significantly among different world regions. The incidence of and mortality from thermal injuries are significantly higher in LMICs than in high-income countries (HICs).^(7)^ Epidemiological studies are crucial to understanding the patient characteristics and outcomes in each particular region. Few epidemiological studies from Latin America, particularly from Uruguay, have been published.^(8-10)^

Uruguay has one specialized unit that treats most adult burn patients (*Centro Nacional de Quemados -* CENAQUE). Patients with burn injuries compromising at least 20% of the total body surface area (TBSA), third degree burns comprising 5% TBSA, burns involving specialized functional areas (hands, face, genitals, joints) and inhalation injuries within Uruguay are usually transferred to this unit. The center treats a predominantly young population, with moderately severe thermal injuries.^(8,9)^

The aim of the present study was to determine the independent risk factors associated with mortality in adult burn patients admitted to this center.

## METHODS

The study was conducted in a specialized burn center (CENAQUE) in a university hospital (*Hospital de Clínicas Dr. Manuel Quintela*, Montevideo-Uruguay). The center has 14 intensive care unit (ICU) beds, two balneotherapy rooms, and one operating room. The center treats only adult burn patients. The treatment strategy has remained unchanged since the opening of the center. During the initial phase, fluid resuscitation is calculated with the Parkland formula. After the first 24 hours, fluid administration is guided mostly by urine output. Closed dressing is performed using silver sulfadiazine as an antimicrobial agent. The coverage strategy consists of early (within 72 hours from admission) excision of full-thickness burns and coverage with autologous or allogenic skin grafts.

A retrospective, observational study was performed by searching the center’s electronic database. Our center uses a custom designed software for medical records, which has been maintained since 1995. Medical parameters must be entered by the attending physicians for all patients upon admission and reviewed before discharge. An external government agency audits those records monthly. The study was approved by the Hospital’s Ethics Committee. All patients with skin burns admitted to the unit since its opening on July 1, 1995 through December 31, 2018 were included in the study. Patients admitted to the center with other diagnoses (degloving injuries, severe cutaneous adverse drug reactions, pemphigus, etc.) were excluded. The demographic data, burn profiles, presence of inhalation injury, length of stay in the unit, mechanical ventilation duration and hospital mortality were studied. Burn severity was evaluated through the Abbreviated Burn Severity Index (ABSI).^(11)^

### Statistical analysis

Continuous variables are expressed as the median (25% - 75% percentile). Categorical variables are expressed as percentages and were compared with the chi-square test. Univariate analysis and multivariate logistic regression were used to identify the risk factors for mortality, expressed as odds ratios (ORs) and 95% confidence intervals (95%CIs). All variables with a p value < 0.2 in the univariate analysis were included in the multivariate logistic regression model. Collinearity between variables in this model was evaluated by analyzing the variance inflation factor. Variables that presented collinearity were excluded from the analysis. The ability of variables that remained significant in multivariate analysis to discriminate ICU survivors from nonsurvivors was determined by receiving operating characteristic (ROC) curves.

The standardized mortality ratio (SMR) was calculated by dividing the number of observed deaths by the number of expected deaths. The latter was calculated by using the ABSI for the probability of death for burn patients. The lethal area fifty percent (LA50), representing the percentage of TBSA associated with a mortality of 50%, was calculated using a probit model. For all comparisons, a p value < 0.05 was considered statistically significant. Data were analyzed using IBM Statistical Package for Social Science (SPSS), version 22.

## RESULTS

**Figure 1:**
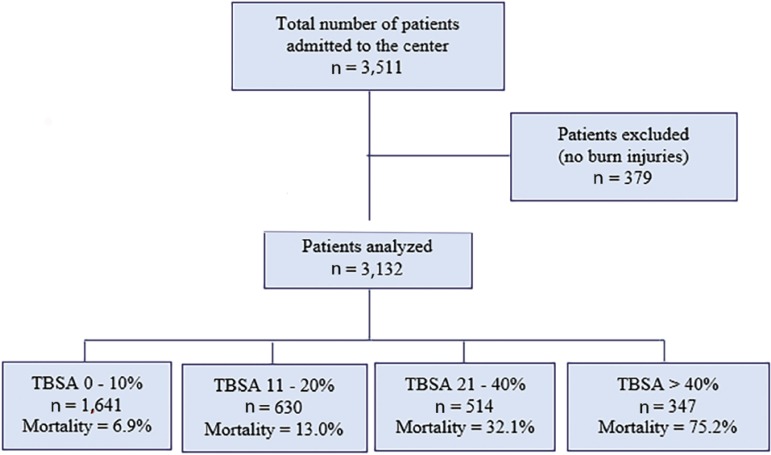
Flowchart of the patients admitted to the center during the study period. TBSA - total body surface area.

During the study period, 3,511 patients were admitted to the center, and 3,132 fulfilled the inclusion criteria (Figure 1 and Table 1). Most of the patients were males (63%), and the median age was 42 (27 - 60) years. The median TBSA burned was 10% (3% - 22%), with 30% of patients presenting a TBSA burned of 20% or more. Flames represented the most common etiology (71.3%), followed by scald injuries (9.3%) and electrical burns (5.5%). The vast majority of burns were caused by domestic accidents (57.0%), followed by accidents occurring at workplaces (9.3%) and suicide attempts (8.7%). The ABSI was 6 (4 - 7). Invasive mechanical ventilation support was required in 60% of the patients for a median duration of 6 (3-16) days. The median length of ICU stay was 17 (7 - 32) days.

**Table 1:** Population characteristics

Characteristic	
Age (years)	42 [27 - 60]
Sex	
Female	63
Male	37
Etiology	
Flames	71.3
Hot liquids	9.3
Electricity	5.5
Chemicals	0.7
Others	13.2
Burn circumstance	
Domestic accident	57.0
Work accident	9.3
Suicide attempt	8.7
Aggression	1.3
Others	23.7
TBSA burned	10 [3 - 22]
ABSI	6 [4 - 7]
Mechanical ventilation duration (days)	6 [3 - 16]
Length of ICU stay (days)	17 [7 - 32]
Mortality	19.9

TBSA - total body surface area; ABSI - Abbreviated Burn Severity Index; ICU - intensive care unit. Results expressed as median [25% - 75% percentiles] or %.

Notably, we detected collinearity between inhalation injury and other variables. Moreover, after reviewing the data, we detected a high risk of overdiagnosis regarding inhalation injury. In many cases, the diagnosis of inhalation injury was made by physicians without experience in treating burn patients based on clinical suspicion before admission to the burn center. Although bronchoscopy was later performed, the diagnosis of inhalation injury was not always updated in the database. Based on these findings, we decided to exclude inhalation injury from the analysis.

**Figure 2:**
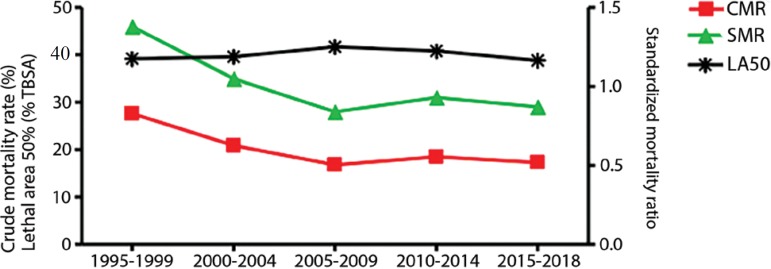
Evolution of crude mortality rate, standardized mortality ratio and lethal area 50 from 1995 to 2018. TBSA - total body surface area; CMR - crude mortality rate; SMR - standardized mortality ratio; LA50 - lethal area 50.

The ICU crude mortality rate (CMR) was 19.9%. The mortality rate was higher for female patients than for male patients (22.3% *versus* 18.5%, p = 0.009). Among the different burn causes, self-inflicted injuries (35.2%) and aggressions (23.1%) had a higher mortality rate than accidental burns (18.2%, p < 0.001). As expected, mortality was significantly higher among patients with more extensive burns (Figure 1, p < 0.001). The LA50 was a TBSA burned of 39.8% (95%CI: 37.4 - 42.6%). The overall SMR was 0.99. Importantly, a significant reduction in crude mortality was observed from 1995 through 2018 (Figure 2, p < 0.001). Moreover, the SMR also decreased since the center’s opening, while the LA50 remained stable (Figure 2).

**Figure 3:**
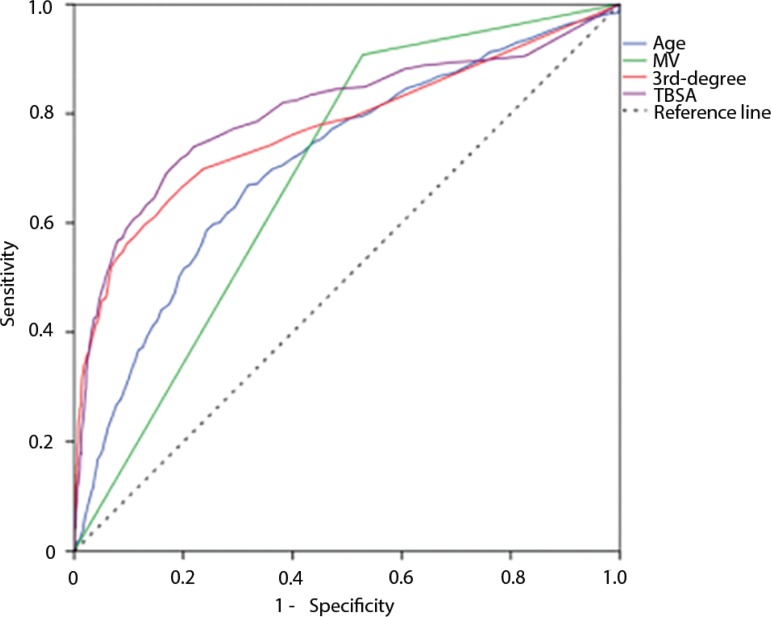
Receiver operating characteristic curves for the risk factors of mortality. MV - mechanical ventilation; 3rd degree - extension of third-degree lesions; TBSA - total body surface area burned.

Univariate analysis found the female sex, older age, suicide attempts, mechanical ventilation, high ABSI, large TBSA burned and extensive third-degree lesions to be the risk factors associated with increased ICU mortality (Table 2). In the multivariate analysis, the need for mechanical ventilation, older age, TBSA burned and extension of third-degree burns remained independent risk factors for mortality. The requirement for mechanical ventilation represented by far, the most important variable associated with mortality (OR 8.80, p < 0.001). The performance of mechanical ventilation (area under the curve [AUC] 0.689, 95%CI 0.665 - 0.714, p < 0.001), age (AUC 0.710, 95%CI 0.681 - 0.738, p < 0.001), TBSA burned (AUC 0.801, 95%CI 0.773 - 0.829, p < 0.001) and extension of third-degree injuries (AUC 0.773, 95%CI 0.744 - 0.803, p < 0.001) to identify nonsurvivors was determined through ROC curves (Figure 3).

**Table 2:** Univariate and multivariate analyses of risk factors for mortality in all patients admitted to the center

Variable	Univariate			Multivariate		
	OR	95%CI	p value	OR	95%CI	p value
Sex (female)	1.27	1.06 - 1.52	0.009			
Age (years)	1.04	1.03 - 1.04	< 0.001	1.07	1.06 - 1.09	< 0.001
Self-inflicted injury	2.44	1.86 - 3.20	< 0.001			
Mechanical ventilation	8.75	6.19 - 12.37	< 0.001	8.80	5.68 - 13.62	< 0.001
ABSI	2.08	1.97 - 2.21	< 0.001			
TBSA burned (%)	1.07	1.06 - 1.08	< 0.001	1.05	1.03 - 1.08	< 0.001
Third-degree lesion (%)	1.09	1.08 - 1.10	< 0.001	1.05	1.03 - 1.07	

OR - odds ratio; 95%CI - 95% confidence interval; ABSI - Abbreviated Burn Severity Index; TBSA - total body surface area.

## DISCUSSION

Burn injuries constitute a major health problem worldwide, particularly in LMICs. There are significant differences in patient characteristics and outcomes among distinct centers and world regions. Knowing the epidemiology and outcomes of a particular center or country is imperative to improving patient care and implementing prevention strategies. This is of particular interest in regions in which previous reports are scarce. The aim of this study was to describe the risk factors associated with mortality in adult burn patients admitted to the CENAQUE. We observed a decreasing trend in crude and standardized mortality for burn patients treated in our center over time. Mechanical ventilation, age, burn area and third degree injury extension were independent factors associated with mortality.

Almost two-thirds of patients admitted to the center were males, and 75% consisted of patients 60 years old or younger. These epidemiologic characteristics are in concordance with those in other studies.^(4,12,13)^ Direct fire represented by far the most frequent etiology. Burn etiology is related to socioeconomic and cultural factors. While hot liquids are frequently the most common agent in HICs, direct fire predominates in LMICs. The burn agent characteristics in our center are similar to those in other reports from Latin America.^(4)^

Our center treated a moderately severe burned population, with an ABSI of 6 (4 - 7). The vast majority of patients had a TBSA burned of less than 20%; in fact, half of the patients had TBSA compromised of 10% or less. Overall, the CMR was 19.9%, with an SMR of 0.99. Importantly, the CMR and SMR decreased continuously since the opening of the unit. This decrease in mortality over time observed in our center is consistent with what has been described by other authors.^(14,15)^ The advances in patient management and supportive care probably explain, at least in part, the improvements in patient outcome. The fact that our center treats the vast majority of adult burn patients in Uruguay helped the multidisciplinary team of the center gain experience. Moreover, educational policies have been implemented since the center’s opening, aiming to improve patient management at the prehospital level and in other facilities. We speculate that these policies may have contributed to the optimization of initial resuscitation and earlier admission to the burn center for more severe patients and are factors that have been associated with better outcomes.^(16,17)^

The need for mechanical ventilation represents a major risk factor for mortality. This is in accordance with other series.^(4)^ Patients requiring mechanical ventilation are especially susceptible to pulmonary infections and to developing acute respiratory distress syndrome, which could be in part responsible for the increased mortality. Older age was associated with a higher mortality, as previously reported.^(6,14)^ This is likely explained by increased frailty and comorbidities in older patients. As expected, mortality as increased as the TBSA burned and third-degree lesion extension increased.^(3,18)^

Our study has limitations that need to be mentioned. First, all data were collected retrospectively. Nevertheless, by using the software designed at our own center, which contains the medical records of all patients included in the study, we consider that the data integrity can be guaranteed. Second, this was a single-center study. Most burns do not fulfill admission criteria to our center and are managed at the emergency department or general ward, so we have no data on an important group of patients. Finally, we could not include data regarding the presence and severity of inhalation injury, infection, use of vasoactive drugs, renal failure or burn agents in the analysis, all of which could be important determinants of patient outcome.

In Uruguay, the CENAQUE where the study was conducted centralizes the treatment of almost all severely burned adult patients in the country. Therefore, for this group of patients, our sample can be considered highly representative of the Uruguayan population.

## CONCLUSION

In concordance with other reports, mechanical ventilation, older age and more extensive burns were associated with decreased survival in Uruguay. More studies are required to improve burn treatment strategies in the region.
